# P-1685. Real-World Outcomes of Chemiluminescence Immunoassay Implementation for Latent Tuberculosis Screening

**DOI:** 10.1093/ofid/ofaf695.1859

**Published:** 2026-01-11

**Authors:** Eli Wilber, Marcos Schechter, Paulina Rebolledo, Sheena Kandiah, Bruce Aldred, Yun F Wang, Susan M Ray

**Affiliations:** Emory University School of Medicine, Atlanta, Georgia; Emory University School of Medicine, Atlanta, Georgia; Emory University School of Medicine, Emory University Rollins School of Public Health, Atlanta, GA; Emory, Atlanta, Georgia; Emory University School of Medicine, Atlanta, Georgia; Emory University School of Medicine, Atlanta, Georgia; Emory University School of Medicine, Atlanta, Georgia

## Abstract

**Background:**

Interferon gamma release assays (IGRAs) are a preferred method of screening for latent tuberculosis infection (LTBI). Recently, a fully automatic chemiluminescence immunoassay (CLIA) became available in the United States with the promise of improving lab workflow. Initial evaluations of this assay have suggested low reproducibility and increased positive results relative to the prior standard of enzyme-linked immunosorbent assays (ELISA). We aimed to characterize the impact of CLIA implementation on our health system.

Table 1

**Figure:**
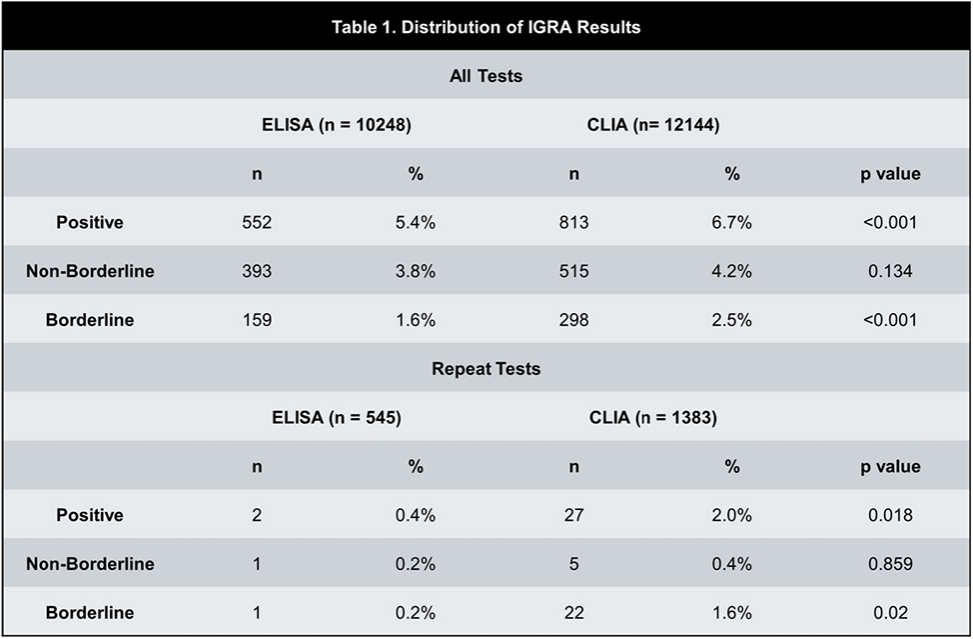

**Figure:**
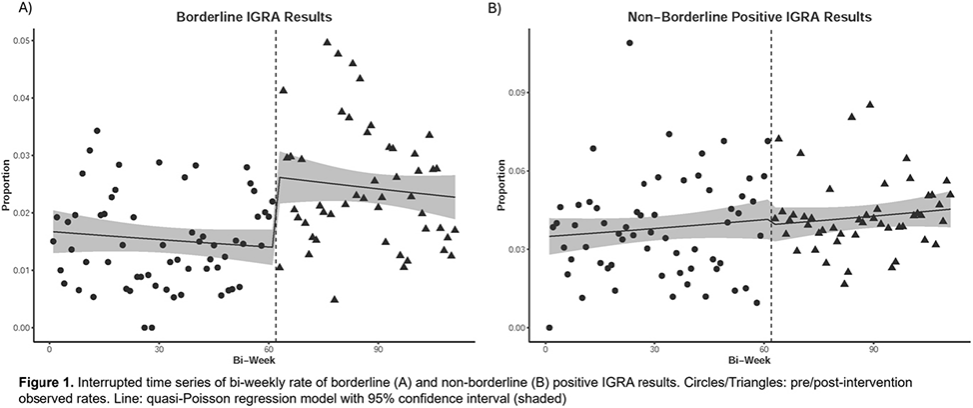

**Methods:**

We conducted a retrospective chart review of all IGRAs conducted at Grady Health System from 1/1/2021 to 3/31/2025 (CLIA implemented on 5/13/2023). We compared the rate of test positivity (TB1 - nil or TB2 - nil >=0.34), borderline positivity (TB1 – nil or TB2 – nil 0.34=< x < 1.0 and neither >=1.0), and non-borderline positivity (positive but not borderline positive) before and after CLIA implementation using the two-proportion z test. An interrupted time series (ITS) analysis using a quasi-Poisson regression model was used to analyze the testing change. For patients with multiple tests, we calculated the rate of seroconversion and the impact on patient management.

**Results:**

22392 IGRAs were performed during the study period (Table 1). Positivity rate (6.7% vs 5.4%; p < 0.001) and borderline positivity (2.5% vs 1.6%; p < 0.001) were higher for CLIA than ELISA. Non-borderline positive results were not significantly different. In ITS analysis (Figure 1), CLIA implementation was associated with a significant level change in the borderline positivity rate but not the nonborderline positivity rate. Of 1928 instances of repeat IGRA testing, 29 were seroconversions of which 27 were borderline positive. Repeat tests on the CLIA were more likely to be a seroconversion (2.0% vs 0.4%, p = 0.018). 15/27 borderline seroconversions were referred for treatment and 8/27 had a therapy modification (e.g. DMARD interruption).

**Conclusion:**

CLIA implementation for IGRAs was associated with increased positivity that is explained by increased borderline positive results that are likely false positives. Diagnostic stewardship measures are needed to mitigate the impact of these results and prevent harm from unnecessary LTBI treatment and/or interruptions of chronic disease therapy.

**Disclosures:**

Eli Wilber, MD, Elsevier: Honoraria|Roche Diagnostics: Honoraria

